# On the origin and development of glioblastoma: multifaceted role of perivascular mesenchymal stromal cells

**DOI:** 10.1186/s40478-023-01605-x

**Published:** 2023-06-24

**Authors:** F. Ah-Pine, M. Khettab, Y. Bedoui, Y. Slama, M. Daniel, B. Doray, P. Gasque

**Affiliations:** 1 Unité de Recherche en Pharmaco-Immunologie (UR-EPI), Université et CHU de La Réunion, 97400 Saint-Denis, France; 2 Service d’Anatomie et Cytologie Pathologiques, CHU de La Réunion sites SUD – Saint-Pierre, BP 350, 97448 Saint-Pierre Cedex, France; 3 Service d’Oncologie Médicale, CHU de La Réunion sites SUD – Saint-Pierre, BP 350, 97448 Saint-Pierre Cedex, France; 4 Service de Médecine d’Urgences-SAMU-SMUR, CHU de La Réunion - Site Félix Guyon, Allée Des Topazes CS 11 021, 97400 Saint-Denis, France; 5 Service de Génétique, CHU de La Réunion - Site Félix Guyon, Allée Des Topazes CS 11 021, 97400 Saint-Denis, France

**Keywords:** Glioblastoma, Mesenchymal stromal cells, Perivascular fibroblasts, Neural crest, Microenvironment, Pericytes, Gliomagenesis

## Abstract

Glioblastoma, *IDH* wild-type is the most common and aggressive form of glial tumors. The exact mechanisms of glioblastoma oncogenesis, including the identification of the glioma-initiating cell, are yet to be discovered. Recent studies have led to the hypothesis that glioblastoma arises from neural stem cells and glial precursor cells and that cell lineage constitutes a key determinant of the glioblastoma molecular subtype. These findings brought significant advancement to the comprehension of gliomagenesis. However, the cellular origin of glioblastoma with mesenchymal molecular features remains elusive. Mesenchymal stromal cells emerge as potential glioblastoma-initiating cells, especially with regard to the mesenchymal molecular subtype. These fibroblast-like cells, which derive from the neural crest and reside in the perivascular niche, may underlie gliomagenesis and exert pro-tumoral effects within the tumor microenvironment. This review synthesizes the potential roles of mesenchymal stromal cells in the context of glioblastoma and provides novel research avenues to better understand this lethal disease.

## Introduction

Glioblastoma (GB), *IDH* wild-type (wt) is the most common and aggressive form of glial tumors, accounting for almost 50% of primary malignant central nervous system (CNS) tumors [1, 2]. It is classified as grade 4 in the World Health Organization (WHO) classification of tumors of the CNS. It belongs to the «adult-type diffuse glioma» family that also includes astrocytoma *IDH*-mutant (WHO grade 2, 3, or 4) and oligodendroglioma, *IDH*-mutant and 1p/19q-codeleted (WHO grade 2 or 3) [1].

As opposed to astrocytoma *IDH*-mutant WHO grade 4 (formerly, GB, *IDH*-mutant), GB, *IDH*wt arises de novo (without preexisting precursor lesion) and typically manifests rapidly after a short clinical history. Despite an aggressive multimodal therapeutic approach, GB *IDH*wt is associated with a dismal prognosis, showing a median survival of 8 months and an overall 5-year relative survival rate of 5.5% [2].

The exact mechanisms of glioblastoma oncogenesis are yet to be discovered. Over the last two decades, extensive and comprehensive molecular profiling of GB has brought new insights into gliomagenesis [3, 4]. The genomic and epigenomic landscape of GB have been thoroughly described, and biological subgroups have emerged, defining three molecular subtypes based on gene expression profiling signatures: proneural, classical, and mesenchymal [3, 5–7]. To date, it has not strongly impacted clinical practice, likely owing to marked intratumoral heterogeneity and differentiation plasticity of GB [8]. However, it has provided new research avenues to understand better the GB pathogenesis, including the identification of the glioma-initiating cell.

Recent studies have led to the hypothesis that GB may arise from neural stem cells (NSC) and glial precursor cells, such as oligodendrocyte and astrocytic precursor cells [9, 10]. In addition, it has been shown that the originating cell lineage is crucial to tumor molecular stratification, independently of the driver mutation that it initially harbors [11, 12]. While glial or neuronal progenitor cells have been suggested to initiate proneural and classical GB, the cellular origin of mesenchymal glioblastoma remains elusive. Studies have described a potential proneural to mesenchymal transition (PMT) that may illustrate the transcriptomic plasticity of GB upon treatment or recurrence. Recently, neural crest (NC)-derived cells have emerged as potential cells of origin in mesenchymal GB [13]. Herein, we discuss perivascular mesenchymal stromal cells (pMSC), also referred to as vascular fibroblasts (vFB), which originate from the NC, as potential candidates for the initiation of GB and their role in GB development [14].

## Glioblastoma

### General characteristics

#### Epidemiology

GB, *IDH*wt is the most common malignant CNS tumor in adults. It accounts for approximately 15% of all intracranial neoplasms and almost 50% of all malignant CNS tumors. It preferentially affects older adults, with a peak incidence in patients aged 55–85 years (median age of 64 years). In the United States of America, GB, *IDH*wt is more common in males compared to females (M: F ratio of 1.58: 1) [1, 2]. To date, the only validated risk factor is ionizing radiation to the head and neck [15, 16]. On the contrary, decreased risk has been observed among individuals with a history of allergies or atopic diseases [16]. Despite a multimodal therapeutic approach that includes surgery, radiotherapy, and chemotherapy, prognosis remains poor, with a 5-year survival rate of 5.5% [2, 17, 18].

#### Definition of GB

GB, *IDH*wt is a diffusely infiltrating high cellular glioma that characteristically shows microvascular proliferation and/or necrosis. As the former term « GB multiforme» suggests, GB morphology has remarkable inter-tumoral and intra-tumoral heterogeneity. Cellular pleomorphism includes small, undifferentiated, spindled, lipidized, granular, epithelioid, and/or giant cells. Secondary structures of Scherer illustrate the different routes that glioma cells can take to invade the brain: 1) the white matter tracts, 2) the vasculature (perivascular satellitosis), 3) the leptomeningeal space and 4) the brain parenchyma.

By definition, GB, *IDH*wt lacks mutations in *IDH1* codon 132 and *IDH2* codon 172. Molecularly, demonstration of *TERT* promoter mutations, *EGFR* gene amplification, and/or a gain of chromosome 7/ loss of chromosome 10 genotype is sufficient for the diagnosis of GB [1].

### GB Molecular pathways

#### PI3K–AKT–mTOR and Ras/MAPK/ERK pathways

The PI3K and MAPK pathways, both activated by receptors tyrosine kinase (RTK), regulate many cellular processes, including cell proliferation. In approximately 90% of IDHwt GB, at least one activating alteration in the PI3K pathway is observed, including alterations of RTK genes, PI3K genes, and *PTEN* [3, 19]. Alterations of RTK genes are common in GB, involving *EGFR* (60%), *PDGFRA* (10–15%), *MET* (2–5%), or *FGFR3* (~ 3%) [3, 5, 20]. PI3-kinase mutations are found in about 25% of GB [3]. In addition, the *NF1* gene, which encodes neurofibromin that functions as a negative regulator of RAS signaling, is deleted or mutated in 10% of cases [3].

#### p14ARF–MDM2–MDM4–p53 pathway

The p53 pathway is altered in a large variety of cancer, including GB. Indeed, up to 90% of GB have an altered p53 signaling pathway, with mutation or deletion of *TP53* in 20–25% of cases [3, 19]. In about 15% of GB, an amplification of *MDM2* or *MDM4* is observed, thus inhibiting p53 [3]. Homozygous deletion of CDKN2A locus, which encodes the p14ARF protein that inhibits MDM2, is detected in about 60% of GB, resulting in an inactivation of the p53 pathway and the pRB pathway (see below) [3].

#### CDK4/6–CDKN2A/B–RB1 cell-cycle pathway

**Fig. 1:**
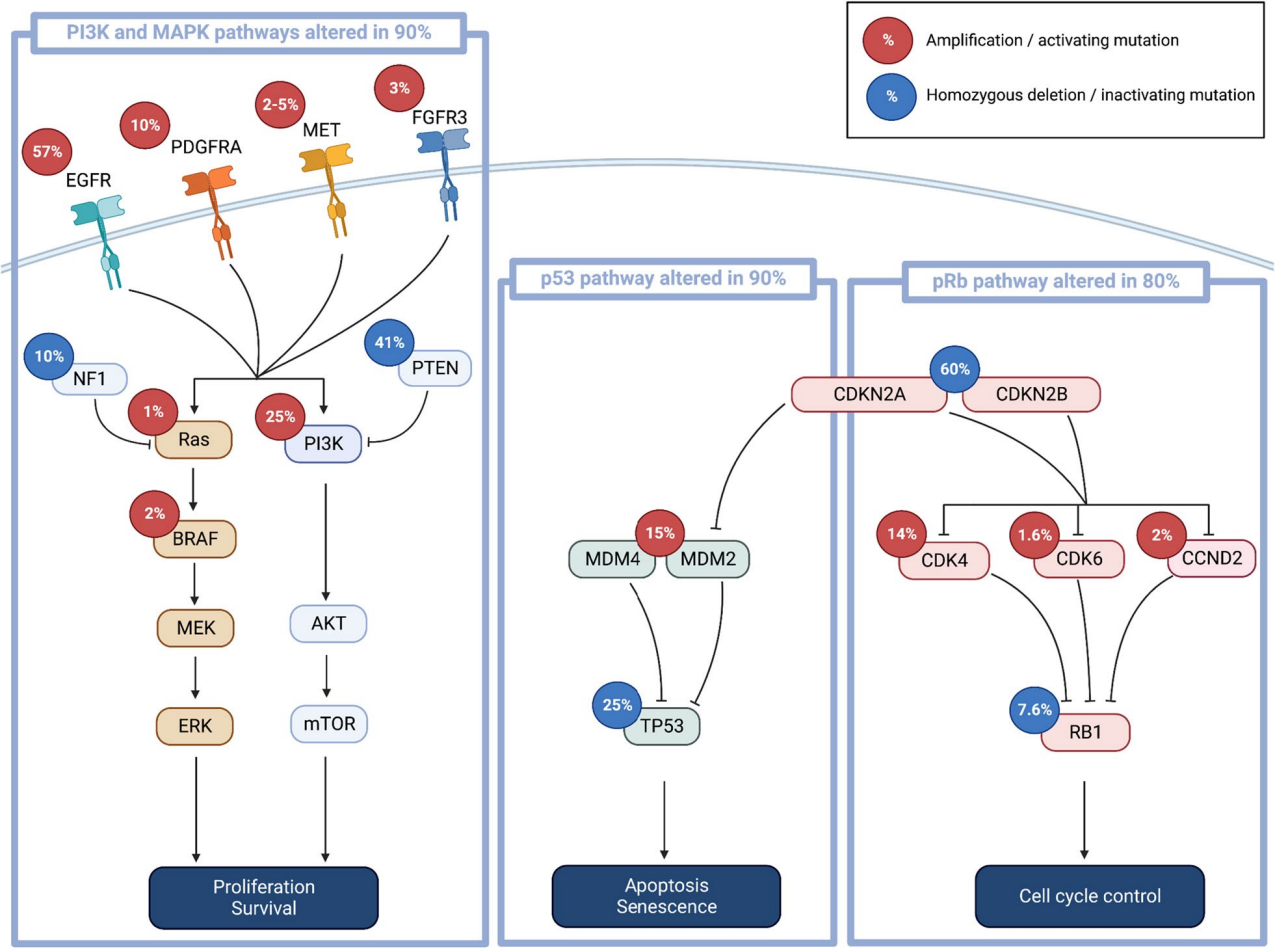
Signaling pathways involved in GB. Alteration rates are summarized for PI3K/MAPK, p53 and pRb regulatory pathways (created with Biorender.com)

The pRB pathway represents a critical cell cycle checkpoint, suppressing cell cycle entry (Fig. 1). CDK4 and CDK6 suppress the downstream inhibition of pRB, allowing the progression from G1 to S phase of the cell cycle. P16, encoded by *CDKN2A*, inhibits CDK4 and CDK6. Up to 80% of GB show at least one alteration of the pRB pathway, including *CDKN2A* deletions, amplifications of *CDK4*/*CDK6,* and inactivating alterations of *RB1* [3, 19].

### GB molecular subtypes and the mesenchymal signature

Gene expression profiling has allowed the classification of GB into three distinct molecular subtypes: proneural, classical, and mesenchymal [3, 5–7]. Initially, this classification was based on the expression profile of 840 genes, but subsequent studies have shown that it can be simplified to rely on just 12 genes with good concordance (Table 1) [21]. However, despite their correlation with distinct genetic aberrations and clinical characteristics, these molecular subtypes have not gained clear significance in clinical practice.

**Table 1:** Glioblastoma molecular subtypes

GB subtypes	Marker genes
Proneural	*P2RX7*, *STMN4*, *SOX10* and *ERBB3*
Classical	*ACSBG1* and *KCNF1*
Mesenchymal	*S100A*, *DAB2*, *TGFB1*, *THBS1*, *COL1A2*, *COL1A1*

Glioblastomas can be classified into three molecular subtypes based on the expression profile of 12 genes

The proneural subtype is characterized by specific genetic alterations, including *IDH1* mutation, *TP53* mutation, *PDGFRA* amplification and/or mutation, and a glioma-CpG island methylator phenotype (G-CIMP) [5, 22]. Notably, both *IDH1* mutations and G-CIMP are considered favorable prognostic factors. However, when excluding *IDH*-mutant tumors, the proneural subtype exhibits the worst prognosis among all subtypes [23].

The classical subtype is characterized by *EGFR* mutation/amplification and *CDKN2A* homozygous deletion.

Accounting for approximately 34% of GB, the mesenchymal subtype displays an expression profile characterized by mesenchymal markers, such as *CHI3L1* and *MET* [5–7]*.* Mesenchymal subtype tumors are predominantly *IDH*wt and G-CIMP- and commonly harbor *NF1* mutation [5, 22, 24]. In addition, they tend to correlate with poor response to radiation therapy and relatively poor outcome [7, 24]. The mesenchymal subtype is also characterized by high levels of angiogenic markers, such as *CD31/PECAM-1*, *VEGF*, *flt1/VEGFR1*, and *kdr/VEGFR2* [6]. Furthermore, it exhibits high expressions of immune-related genes, particularly proinflammatory genes and immunosuppressive genes [3, 5, 6, 25]. Several of these genes are involved in the recruitment of monocytes/macrophages (CSF-1, CCL2, CCL-22, TREM1, and TREM2) and in the macrophage-polarization towards an immunosuppressive M2-phenotype (CD163, CD204) [25]. Notably, the mesenchymal subtype shows enrichment of macrophages and microglial cells, constituting the largest stroma cell population in GB [26–28].

It is important to note that while an initial neural subtype was described in this classification, it was later considered to be the result of contamination with normal cells [5, 21, 23].

### The origin of GB

**Fig. 2:**
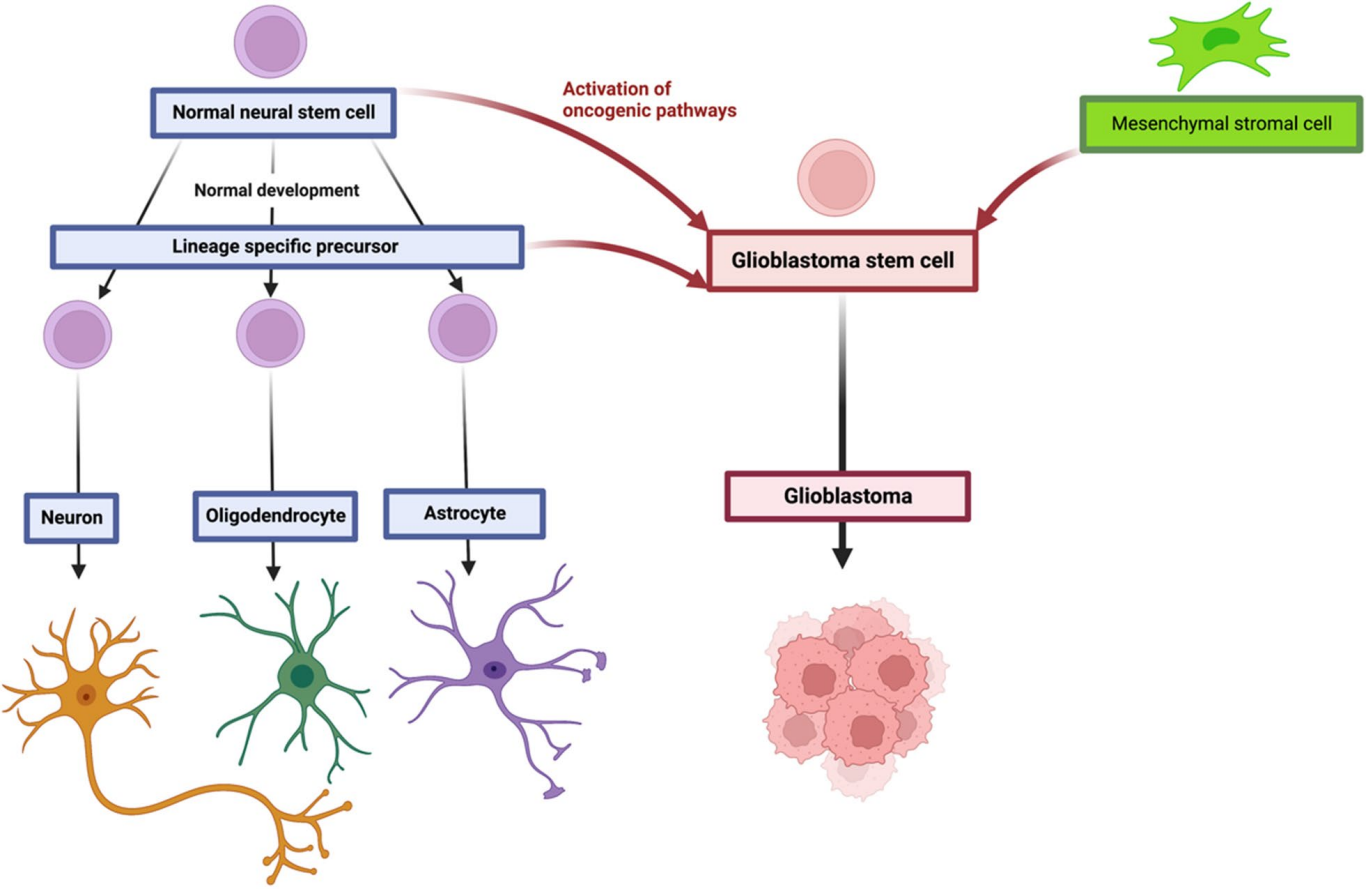
The origin of glioblastoma. During normal embryonic development and in the adult brain, normal neural stem cells generate glial and neuronal cells. Glioblastoma stem cells may arise from neural stem cells and/or glial precursor cells through the activation of oncogenic pathways. They may also originate from neural crest (NC)-derived, pMSC. During development, the NC arises from the neural tube and its component cells migrate and invade virtually all tissues, giving rise to numerous differentiated cells, such as pMSC, melanocytes, chondrocytes, peripheral neuronal and glial cells, thyroid C cells, and adrenergic cells (created with Biorender.com)

The exact cell of origin of GB has yet to be definitively identified. Several CNS cell types within the CNS, including neural precursor cells (NPC), oligodendrocyte precursor cells (OPC), and astrocytic precursor cells (APC), have been proposed as potential candidates for initiating GB (Fig. 2) [9, 29, 30]. Moreover, emerging evidence indicates that the cell lineage plays a crucial role in determining the molecular subtype of GB. Indeed, the introduction of identical driver mutations in different precursor cells leads to the development of distinct molecular subtypes [11, 12]. Studies have demonstrated that neural stem cells (NSC) in the subventricular zone carry the driver mutations responsible for GB, suggesting them as a potential cell of origin [10, 31, 32]. Further supporting this notion, single-cell RNA-sequencing (scRNAseq) studies have identified profiles resembling NPC, OPC, and APC, providing evidence for a neuronal/glial origin of GB [8, 33]. However, the cellular origin of GB with mesenchymal features, despite the well-described mesenchymal transcriptomic profile, remains elusive. The hypothesis of pMSC as GB-initiating cells will be discussed further (Fig. 2).

**Fig. 3:**
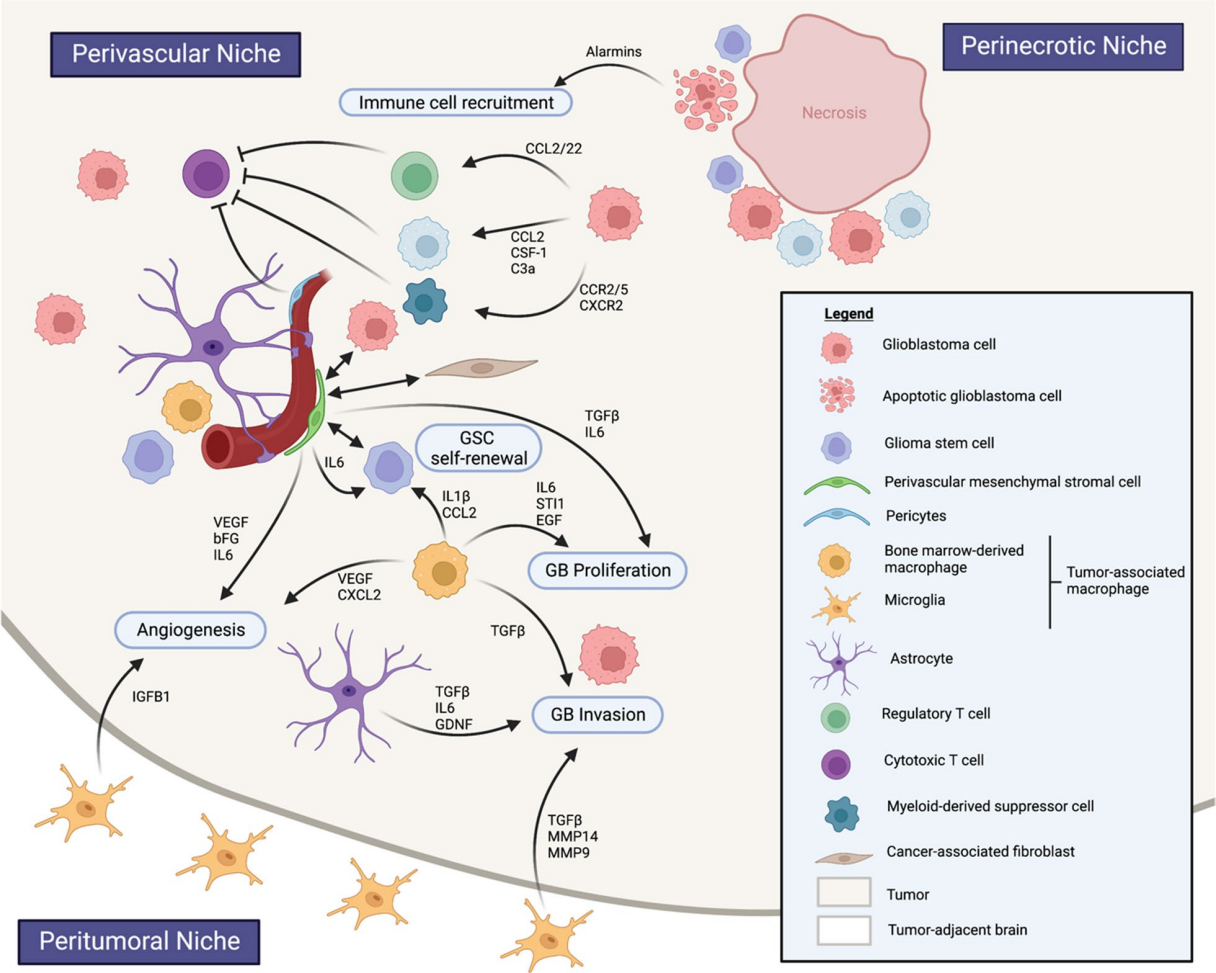
Glioblastoma tumor micro-environment. GB TME is compartmentalized in perivascular, perinecrotic and peritumoral niches. Tumor-associated macrophages (Bone marrow-derived macrophages and microglia) and mesenchymal stromal cells play key roles in supporting GB proliferation, invasion and angiogenesis (created with Biorender.com)

To support the hierarchical development model, CD133+ glioma stem cells (GSC) have been identified within GB tumors. These GSC exhibit remarkable proliferation capacity, self-renewal abilities, and differentiation potential [34, 35]. They are characterized by the expression of CD133, OCT4, CD44, nestin, and SOX2, although a specific marker exclusive to GSC has not yet been identified [34, 36, 37]. GSC are described as slow-dividing or quiescent cells that reside in protective microenvironments called GSC niches, contributing to intratumoral heterogeneity and therapy resistance [34, 38, 39]. GSC preferentially reside in the perivascular niche, interacting with endothelial cells in intricate bidirectional crosstalk, and in the perinecrotic niche (Fig. 3) [40].

## GB immune microenvironment

GB is a highly complex tissue composed of tumor cells and their surrounding microenvironment which supports tumor growth through a permissive neighborhood. The tumor microenvironment (TME) consists of cells (including immune cells, vascular cells, glial and neuronal cells, and stem cells), soluble factors, signaling molecules, and an extracellular matrix. It is a dynamic milieu considered to play an active role in tumorigenesis through reciprocal communication with cancer cells [41]. GB TME is compartmentalized in tumor niches which are critical regions where interactions between cancer cells and host cell populations are promoted (Fig. 3).

### Tumor vasculature

One of the main features of GB is microvascular proliferation. The tumor vasculature plays a crucial role in supporting tumor growth through various mechanisms, including:

- *Angiogenesis* This is the primary process involved in GB vascularization, triggered by the release of pro-angiogenic factors, such as vascular endothelial growth factor (VEGF), by tumor cells.
- *Vessel co-option* Tumor cells possess the ability to migrate along existing blood vessels, enabling invasion of the brain through vascular routes [42].
- *Vasculogenesis* This process involves the recruitment of endothelial cell progenitors derived from the bone marrow, contributing to the assembly of neo-vessels [43].
- *Transdifferentiating process* GSC demonstrate the capacity to differentiate into tumor-derived endothelial cells, a phenomenon known as the transdifferentiating process [44, 45].
- *Vascular mimicry* This refers to the presence of vascular structures within the tumor that result from GSC differentiating into vascular smooth muscle cells (vSMC) or pericytes (PC) [46, 47].

### Macrophages

Immune cells may represent up to 50% of the GB tumor bulk [48]. Among these immune cells, tumor-associated macrophages (TAM) are the predominant population and are characterized by their origin, localization, and functions, encompassing both microglia and bone marrow-derived macrophages (BMDM) [49–51]. Notably, BMDMs represent approximately 85% of TAM and are primarily found in perivascular regions within the tumor, while microglia are localized in peri-tumoral areas [51, 52].

TAM can exhibit different activation states depending on environmental cues, polarizing into either type I response (M1 TAM) or type II response (M2 TAM) through classical or alternative activation, respectively. M1 TAM promote inflammation by producing pro-inflammatory cytokines, such as IL- 12, IL-1β, TNF-α, IL-6, and IL-23, while M2 TAM suppress inflammation by producing ARG1, IL-10 and IL-4 [53]. Initially, once recruited within the GB TME, TAM were considered to polarize toward an M2-like phenotype that promotes invasion, angiogenesis and immunosuppression [54–56]. However, recent studies have revealed that TAM encompass a dynamic entity that includes antitumoral M1-like, pro-tumoral M2-like, and non-polarized M0 phenotypes [27, 57].

Regarding GB molecular subtypes, mesenchymal GB exhibit higher *AIF1* expression (encoding for IBA1), a marker associated with TAM [28]. These findings is consistent with previous studies showing increased infiltration of TAM in *NF1*-altered GB [7, 58]. In addition, it has been suggested that PMT was associated with increased TAM infiltration [24].

Functionally, TAM play a pivotal role in gliomagenesis through complex cross-talk with tumor and TME cells, contributing to tumor progression, immunosuppression, and cerebral edema [59]. TAM release factors such as TGFβ, IL-1β, IL-6, stress-inducible protein 1 (STI1), and epidermal growth factor (EGF) that stimulate tumor growth and invasion (Fig. 3) [60–63]. In addition, their immunosuppressive role includes the recruitment of CD4+ /FOXP3+ T regulatory (Treg) cells and myeloid-derived suppressor cells (Fig. 3) [64, 65]. Furthermore, due to their perivascular localization, TAM have been investigated for their involvement in cerebral edema. Studies have shown that dexamethasone, commonly used for the management of cerebral edema, inhibits TAM production of IL-1β, and genetic ablation of IL-1α/β or IL-1β in a murine GB model or the administration of a potential IL-1β inhibitor (Sulfasazaline) reduces cerebral edema [66, 67]. The potential role of TAM in vasogenic cerebral edema underscores the need for further investigations into the complex interaction of TAM with the components of the blood–brain barrier (BBB).

These findings point out the crucial role of the perivascular niche in gliomagenesis, by promoting angiogenesis, modulating the immune response, supporting tumor cell invasion, and providing a stem cell niche. Within this niche, pMSC are also present, and their role in GB development and progression will be discussed below.

### Perivascular mesenchymal stromal in the CNS

#### Definition

First identified in the bone marrow (BM) and termed colony-forming unit fibroblasts (CFU-F), MSC are characterized in vitro by a spindle-shaped, fibroblast-like, plastic-adherent appearance [68, 69]. They are multipotent progenitor cells that have the ability to differentiate into adipocytes, chondrocytes, and osteoblasts [70–72]. Their multipotency has raised much interest in tissue engineering research for using culture-expanded MSC to replace injured or damaged mesenchymal tissue [73]. MSC express CD105 (Endoglin), CD73, and CD90 (Thy1) and lack the expression of CD45, CD34, CD14 or CD11b, CD79a or CD19, and HLA-DR [70, 71, 74]. Other markers, such as CD140b (PDGFRB), CD271 (Low-affinity NGF receptor), CD146 (Muc18), and CD248 have been suggested to identify MSC [75–78] but these markers are equally expressed by PC. As the concept of MSC was initially defined as a multipotent cell residing within the BM, it has evolved over the years to a wide concept that includes multipotent perivascular cells of any organ, including the CNS, as discussed below.

#### Origin of pMSC and PC in the CNS vasculature

MSC in adult tissues have two main embryonic origins, deriving either from the mesoderm or the NC [79–83]. During embryogenesis, MSC migrate along vessels and then reside in the perivascular niche of all adult tissues, adopting similar features to that of PC [80, 84].

The term ‘PC’ is often used in the literature to refer to microvascular periendothelial cells [85]. The accepted definition of a PC is a cell that is embedded within the vascular basement membrane, as observed by electron microscopy. However, because ultrastructural analyses are impractical, most published papers may not differentiate PC from other periendothelial cells, including vSMC and pMSC [14, 86–91]. It is now clear that different cell types exist in the periendothelial compartment but accurately identifying their phenotypes remains a challenge. PC cannot be definitively identified and distinguished from vSMC or pMSC using a single molecular marker. Commonly applied markers or genes (Table 2), such as NG2/Cspg4, CD13/Anpep, and desmin, are not specific and their expression is not stable, particularly in disease conditions. Other markers, such as CD248 (endosialin) and CD90 (Thy-1), are highly expressed by PC but recent investigations revealed that they are also expressed by pMSC, especially in the context of GBM [86]. In addition, it has been demonstrated that PC, originally defined by their vascular mural localization, have the same osteogenic, adipogenic, and myogenic potential as MSC and also express surface markers of MSC, such as CD44, CD73, CD90, and CD105 in vitro [80, 92].

**Table 2:** Identification and markers (*genes*) of endothelial and perivascular cells in the CNS

Cell types	Markers	Comments	Reference
Endothelial cells	**CD31**	PECAM-1, cell adhesion	[86]
	**CD93**	CD248 family member	[86, 95]
	CLDN5	Claudin 5	[86]
	CDH5	Cadherin 5, also expressed by CNS fibroblasts	[86]
Pericytes (PC)	**PDGFRβ**	Receptor for platelet derived growth factor	[85]
	**NG2**	Encoded by Chondroitin sulfate proteoglycan 4 *CSPG4*. Also expressed by oligodendrocyte progenitor cells	[85]
	Desmin		[85]
	CD13	Aminopeptidase N	
	**CD248**	Endosialin (TEM-1), highly expressed in glioma (GBM) (PC > pMSC)	[85, 96, 97]
Vascular smooth muscle cells (vSMC)	PDGFR**β**	Levels in PC > vSMC	[98]
	NG2		[98]
	Desmin	Muscle class III intermediate filament	[98]
	CD13		
	RGS5	Regulator of G protein signaling 5 GTPase activating protein	[85, 99]
	CD146	Melanoma cell adhesion molecule (MCAM)	[85, 99]
	**αSMA**	Alpha smooth muscle cell actin encoded by *ACTA2*. Level of expression in vSMC > > PC	[85]
	**TAGLN**	Trangelin, smooth muscle protein 22 alpha (SM22)	[85]
pMSC fibroblast-like	PDGFR**β**		
	**PDGFRα**		[86]
	CD13		
	COL1A1	pMSC also express high levels of COL1A2, COL3A1, COL4A1. Expressed by PC (less than 2%)	
	**LAMA-1**	Laminin subunit alpha 1, also expressed by epithelial cells	[86]
	LUM	Lumican	[86]
	DCN	Decorin	[86]
	MPZL2	Myelin protein zero-like 2, adhesion	[86]
	SRPX2	Sushi Repeat Containing Protein X-Linked 2	[86]
	FN	Fibronectin, also expressed by PC / scar tissue	[100]
	**FBLN1**	Fibulin-1, Type I Hu pMSC/fibroblast	[14]
	**CEMIP**	Cell migration-inducing protein, Type II Hu FB	[14]
	**KCNMA1**	Potassium channel, Type III Hu pMSC/Fib	[14]
Macrophages (M)	CD11b / CD18	Complement receptor type 3 involved in phagocytosis of host cell debris	[87, 101]
Border-associated M (BAM)	**CD163**	Scavenger receptor, M2 antiinflammatory	[102]
	CD206	Mannose receptor	[88]
	LYVE1	Hyaluronan receptor	[88]
Astrocytes (AS)	**GFAP**	Glial fibrillary acidic protein	[87]

Several markers are shared between the different subsets of periendothelial cells while others (in bold) are restricted (but not exclusive) to specific subsets. TEM1: Tumor endothelial marker 1

Recent studies utilizing cell lineage tracing and single-cell RNA sequencing experiments have provided insights into the role of pMSC cells in the CNS. Garcia et al. have identified 11 cell subtypes within the human CNS vasculature, including three distinct subtypes of pMSC (referred to as vFB in this study), with specific markers (Table 2) [14]. Type I pMSC in humans appear to be primarily involved in extracellular matrix (ECM) organization and fibrosis, while type III cells express various growth factors, including VEGFA. Interestingly, the gradient of gene expression from type I to type II pMSC was continuous with a subpopulation of pericytes, suggesting a potential lineage from type I to type II to PC [14]. Two of the pMSC subtypes align with the subtypes previously identified in mice by Vanlandewijck et al. (referred to as vFB in this study, type I and II) [86]. Similar findings were observed in a zebrafish study, which demonstrated the stem cell potential of pMSCs to transdifferentiate into PC [93].

These findings substantiate the affiliation of PC and pMSC, which are also referred to vFB, within a continuum of differentiation [72, 94].

#### Physiological functions of PC and pMSC in the CNS.

**Fig. 4:**
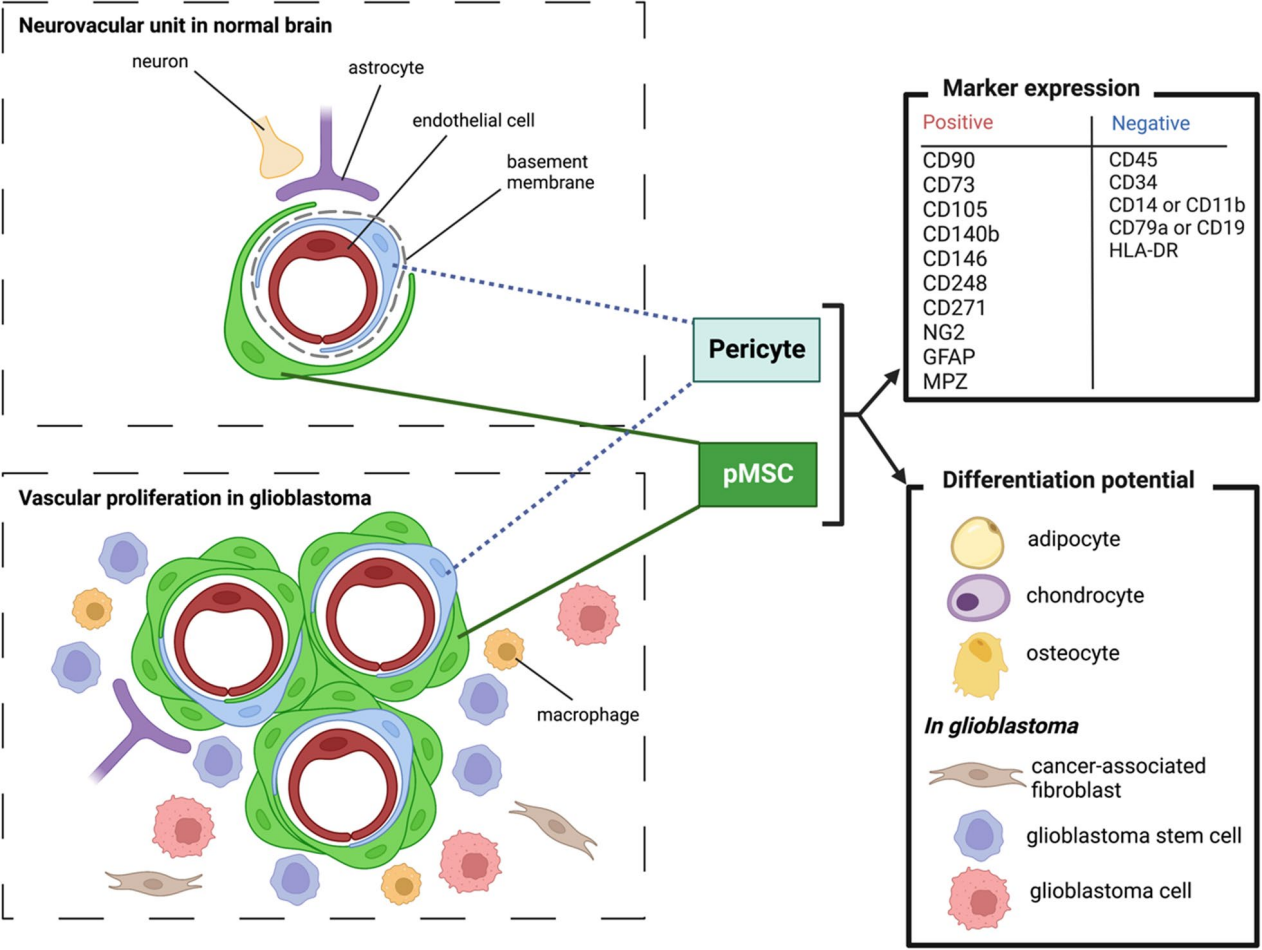
Perivascular mesenchymal stromal cells (pMSC) in normal brain and in glioblastoma. In normal brain, pMSC form the neurovascular unit, together with endothelial cells, astrocytes, and neurons. The neurovascular unit supplies nutrients and oxygen through the blood brain barrier. In glioblastoma (GB), resident pMSC and glioma stem cell-differentiated pMSC participate in vascular proliferation. Leaving the vessel, pMSC may give rise to GB stem cells, GB cells, and cancer-associated fibroblasts. (MPZ: Myelin P zero) (created with Biorender.com)

The close association between PC and endothelial cells contributes to the formation of the BBB, the maintenance of vascular stability, and the regulation of vascular tone [103–105]. Other functions have been described, including a role in angiogenesis and immune regulation properties, making pMSC key players in brain homeostasis and disease. Together with endothelial cells, astrocytes and neurons, they form the neurovascular unit that supplies nutrients and oxygen through the BBB and provides an optimal environment for NSC (as well as GSC) homing and proliferation (Fig. 4) [106, 107].

#### Injury repair

Many studies have demonstrated the ability of MSC to differentiate toward a neuronal/glial phenotype in vitro and therefore, have suggested a potential role of MSC in brain repair [108–112]. However, while transplantation of MSC in brain and spinal cord injury models tends to improve the functional outcome, the transformation of MSC into neurons/glial cells in vivo is rare and partly results from the fusion of MSC with brain cells [113–116]. Consequently, it has been suggested that the role of MSC in brain injury mostly relies on their immune regulation properties rather than their neuronal differentiation ability. In fact, this paradigm shift in which MSCs exert healing effects not through their differentiation abilities but rather through their immune modulation functions, has been observed in many therapeutic contexts [117–120].

Recent findings suggest that pMSC and PC may have a unique ability to monitor the microenvironment of injured tissues. Indeed, it has been demonstrated that they secrete a large number of chemokines, cytokines, and other soluble factors [120–123]. Their role in immune regulation has initially been highlighted by the observations of prolonged skin graft survival, improvement in severe graft-versus-host disease, and therapeutic effects in an experimental autoimmune encephalomyelitis mouse model [124–126]. Indeed, MSC can modulate effector T-cell activation and proliferation, directly through soluble factors or indirectly by controlling the activity of regulatory T-cells (Treg). MSC are also able to control the proliferation and the activation of monocytes/macrophages, natural killer T-cells, dendritic cells, B-cells, and neutrophils, by the secretion of soluble factors such as IFN gamma, nitric oxide (NO), indoleamine 2,3-dioxygenase (IDO), prostaglandin E2 (PGE2), TGFβ, and IL10 [127, 128]. Consequently, MSC have a major role in the coordination of healing responses and the prevention of autoimmunity [119, 127–129].

Scar formation is a ubiquitous healing mechanism that is preserved throughout the CNS. Initially, the CNS scar has been referred to as the glial scar as a whole. The glial scar predominantly consists of reactive astrocytes and proteoglycans (heparan sulphate proteoglycan, dermatan sulphate proteoglycan, keratan sulphate proteoglycan and chondroitin sulphate proteoglycan) that stabilize injured CNS tissue by modulating the inflammatory response, yet prevent tissue regeneration [130–132]. The glial scar circumscribes the lesion core where the inflammatory response leads to a fibrotic scar, composed of immune cells, fibroblasts, fibronectin, collagen and laminin [133]. It is commonly accepted that fibroblasts are absent in the CNS parenchyma and it has been suggested that they are restricted to the vascular and meningeal niches [134]. Several studies have underscored the role of pMSC and PC in generating the fibrotic scar in the CNS [100, 135–137]. In response to spinal cord injury, PC proliferate locally and give rise to myofibroblasts, generating the fibrotic scar [100]. A rapid pMSC/PC loss after cerebral ischemia in human stroke has been observed, with subsequent proliferation of resident PDGFRβ + CD13 + stromal cells that transform to αSMA + CD105 + myofibroblasts [135]. These findings suggest the critical role of the endothelial cell-pMSC/PC interaction to maintain pMSC and PC in a quiescent state to prevent fibrosis.

#### pMSC in GB

In GB, pMSC can be recruited either from local brain sources, in the perivascular niches, or from the BM by MSC homing to the GB TME [138, 139]. pMSC may also result from GSC differentiation. As discussed above, GSC predominantly reside in perivascular niches and interact with endothelial cells in a bidirectional manner [40]. First reports have suggested that GSC may transdifferentiate into endothelial cells but it has been shown that endothelial cells do not harbor molecular alterations of GB [44, 140–142]. In addition, the ability of GSC to undergo mesenchymal differentiation has raised the hypothesis of GSC transdifferentiating into pMSC rather than endothelial cells [143, 144]. Furthermore, it has been demonstrated that GSC generate PC, which may carry the same genetic alterations of GB, such as *EGFR* amplification, chr 10 loss and *PTEN* loss [145].

**Fig. 5:**
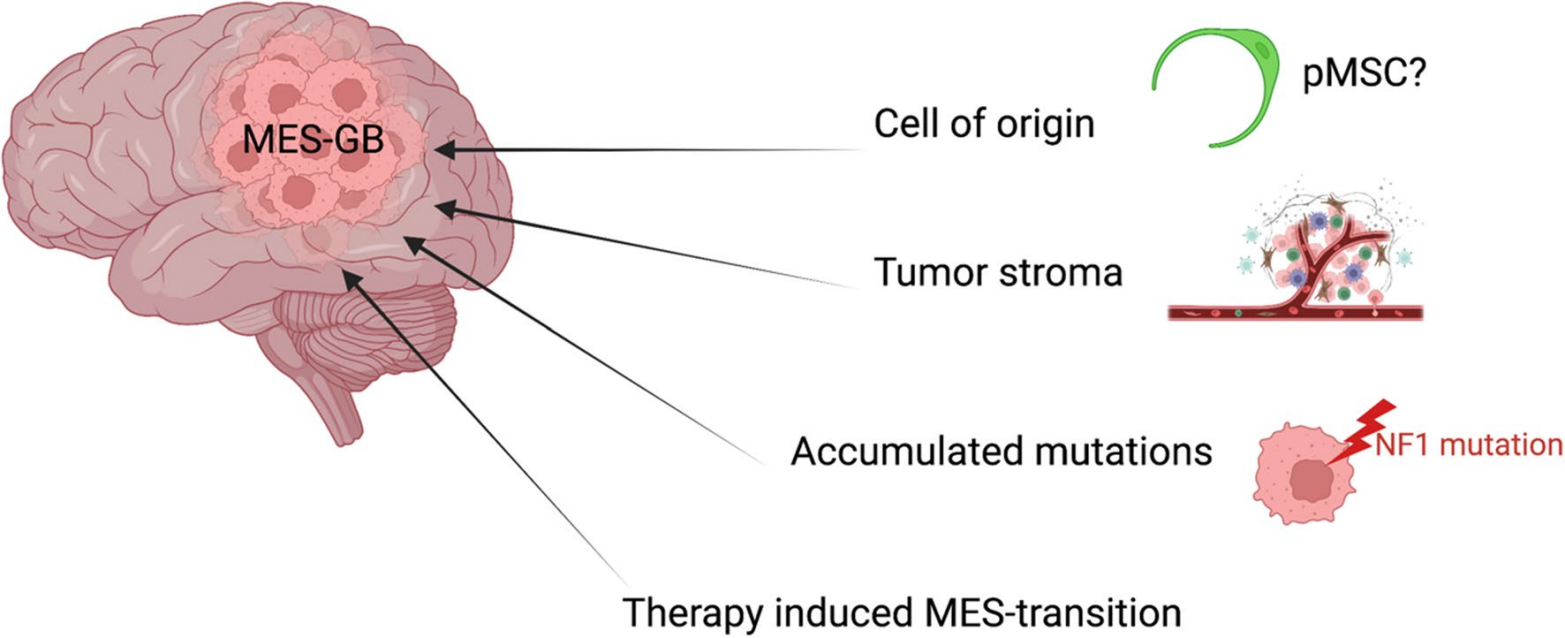
Origin of mesenchymal glioblastoma subtype (created with Biorender.com)

As discussed above, the origin of mesenchymal GB remains elusive and until recently, an alternative non-neural progenitor cell has not been explored. Indeed, deep scRNAseq of GB progenitor cells uncovered two principal cell-lineage profiles, NC perivascular and radial glia (and its progenies) [13]. Consistently, introducing driver mutations in perivascular cells was sufficient to initiate brain tumors in vivo. In addition, it has been shown that GB of a perivascular lineage represent 44% of the mesenchymal GB subtype and showed significant poorer survival than those of radial glia-lineage [13]. These results suggest that the mesenchymal signature results, at least partially, from pMSC transformation. Indeed, the mesenchymal subtype can be induced by other factors such as the influence TME, the accumulation of mutations in tumor cells (particularly *NF1* mutation) and the therapy-induced mesenchymal transition (Fig. 5) [146].

Several studies have demonstrated the involvement of PC and pMSC in GB tumor vasculature development through a vascular mimicry mechanism [46, 47, 139, 147, 148]. It has also been shown that pMSC overexpress several proteins involved in the promotion of tumor angiogenesis, including CSPG4/NG2, CRYAB, CNN1, CALD1, and VASP, and secrete high levels of angiogenic factors such as SDF-1/CXCL12 and HGF [149].

The role of pMSC in immune regulation during GB progression was demonstrated by the high levels of anti-inflammatory cytokines (IL-10 and TGFβ) detected in vitro and in vivo in pMSC (referred as to PC in this study) that interact with GB cells [150]. In contrast, after activation by GB cells, pMSC did not produce proinflammatory cytokines, such as IL-1, IL-23, and IL-12 [150]. These observations suggest an immunosuppressive response of pMSC to interaction with GB cells.pMSC also have a tumor growth-enhancing and tumor invasiveness-increasing role [138, 151]. It has been demonstrated that pMSC secrete TGFβ1, stimulating GB cell proliferation and viability through paracrine effect [152]. pMSC are also capable of enhancing GB cell proliferation under direct cell–cell contact, independently of TGFβ1 levels, in vitro and in vivo [152]. Similarly, it has been shown that pMSC secrete IL-6, increasing proliferation and self-renewal of GSC in vitro and enhancing GSC tumorigenicity in vivo (Fig. 3) [153].

Studies have isolated two subpopulations of pMSC (CD90^high^ pMSC and CD90^low^ pMSC) and have described specific roles in GB progression [154, 155]. It has been observed that CD90^low^ pMSC are more abundant than CD90^high^ pMSC and that CD90^low^ pMSC contribute to angiogenesis and CD90^high^ pMSC promote GB cell growth both in vivo and in vitro [155, 156]. Indeed, CD90^low^ pMSC were shown to produce higher levels of angiogenic factors, such as VEGF, bFGF and IL-6, and CD90^high^ pMSC to produce higher levels of growth factors, such as SDF-1α, CCL5 and MMP9 [155].

Perivascular and intratumoral cells that co-express PDGFRβ and fibroblast activation protein α (FAP), a common marker used to identify cancer-associated fibroblasts (CAF), were identified in GB [157]. Proteomic quantitative analysis has also demonstrated that pMSC expressed high levels of CAF markers, such as CD146, S100A4/FSP1, nestin, and NG2 [149]. These findings suggest that pMSC, mirroring their transition to myofibroblasts in the context of fibrotic scar, may give rise to CAF that support tumor progression with the GB TME, as described in other solid cancers [158, 159].

## Concluding remarks

pMSC exert pro-tumoral effects, promoting angiogenesis, tumor proliferation and invasiveness, and immunosuppression, in agreement with the observation that increased percentages of pMSC within high-grade gliomas are associated with worse clinical outcome [160]. Recent studies suggest that, in addition to their activities to support GB growth, pMSC may be the cell of origin of GB, particularly the mesenchymal GB subtype [13]. This alternative paradigm provides exciting new research avenues to characterize pMSC in the context of GB and understand better the gliomagenesis.

## Data Availability

Not applicable

## Abbreviations

ACSBG1: Acyl-CoA synthetase bubblegum family member 1
AIF1: Allograft inflammatory factor 1
APC: Astrocytic precursor cells
BBB: Blood–brain barrier
BMDM: Bone marrrow-derived macrophage
CAF: Cancer-associated fibroblast
CALD1: Caldesmon
CCL: C-C motif chemokine ligand
CDK4: Cyclin-dependent kinase 4
CDK6: Cyclin-dependent kinase 6
CDKN2A: Cyclin-dependent kinase inhibitor 2A
CFU-F: Fibroblastic colony forming units
CHI3L1: Chitinase 3 like 1
CNN1: Calponin 1
CNS: Central nervous system
COL1A1: Collagen type I alpha 1
COL1A2: Collagen type I alpha 2
CRYAB: Crystallin alpha B
CSF-1: Colony stimulating factor 1
DAB2: Disabled-2
EGF: Epidermal growth factor
EGFR: Epidermal growth factor receptor
ERBB3: Erb-b2 receptor tyrosine kinase 3
FAP: Fibroblast activation protein-α
FGFR3: Fibroblast growth factor receptor 3
FOXP3: Forkhead box P3
GB: Glioblastoma
G-CIMP: Glioma-CpG island methylator phenotype
GSC: Glioma stem cell
HGF: Hepatocyte growth factor
IBA1: Ionized calcium binding adaptor molecule 1
IDH: Isocitrate dehydrogenase
IDO: Indoleamine 2,3-dioxygenase
IFN: Interferon
IL: Interleukin
KCNF1: Potassium voltage-gated channel modifier subfamily F member 1
MAPK: Mitogen-activated protein kinase
MDM2: Mouse double minute 2
MDM4: Mouse double minute 4
MET: Mesenchymal-epithelial transition factor
MMP9: Matrix metallopeptidase 9
NF1: Neurofibromatosis type 1
NG2: Neural/glial antigen 2
NO: Nitric oxide
NPC: Neural precursor cells
NSC: Neural stem cells
OCT4: Octamer-binding transcription factor 4
OPC: Oligodendrocyte precursor cells
P2RX7: Purinergic receptor P2X 7
PC: Pericytes
PDGFR: Platelet-derived growth factor receptor
PECAM1: Platelet endothelial cell adhesion molecule 1
PGE2: Prostaglandin E2
PI3K: Phosphoinositide 3-kinase
pMSC: Perivascular mesenchymal stromal cell
PMT: Proneural to mesenchymal transition
pRB: Retinoblastoma protein
S100A: S100 calcium-binding protein A
scRNAseq: Single-cell RNA-sequencing
SDF-1: Stromal cell-derived factor 1
SMA: Smooth muscle actin
SOX10: SRY-box transcription factor 10
SOX2: SRY-box transcription factor 2
STMN4: Stathmin 4
TAM: Tumor-associated macrophage
TERT: Telomerase reverse transcriptase
TGFβ: Transforming growth factor beta
THBS1: Thrombospondin 1
TME: Tumor microenvironment
TREM1: Triggering receptor expressed on myeloid cells 1
TREM2: Triggering receptor expressed on myeloid cells 2
VASP: Vasodilator-stimulated phosphoprotein
VEGF: Vascular endothelial growth factor
VEGFR: Vascular endothelial growth factor receptor
vFB: Vascular fibroblast (or perivascular fibroblast)
WHO: World Health Organization
